# Mortality risk effects of ozone and meteorological factors: a 10-year time-series study

**DOI:** 10.3389/fpubh.2026.1742521

**Published:** 2026-02-18

**Authors:** Na Cao, Xiaojuan Yang, Yifei Chen, Lifang Zhao, Shuai Guo, Rui Li, Guiming Zhu, Lin Ma, Zhihong Zhang

**Affiliations:** 1 Department of Environmental Health, School of Public Health, Shanxi Medical University, Taiyuan, China; 2 Yellow River Basin Ecological Public Health Security Center, Shanxi Medical University, Taiyuan, China; 3 MOE Key Laboratory of Coal Environmental Pathogenicity and Prevention, Shanxi Medical University, Taiyuan, China; 4 Shanxi Center for Disease Control and Prevention, Taiyuan, China; 5 Department of Health Statistics, School of Public Health, Shanxi Medical University, Taiyuan, China

**Keywords:** generalized additive model, health risk assessment, interaction, meteorological factors, mortality, ozone

## Abstract

**Background:**

Tropospheric ozone (O₃) is increasingly becoming the dominant urban air pollutant in China, posing significant public health risks that are exacerbated by meteorological conditions. A clear understanding of how O₃-related health effects are modified by atmospheric factors is crucial for targeted risk mitigation.

**Methods:**

This ten-year time-series study (2013–2022) was conducted in Taiyuan, China. We analyzed data on daily O₃ concentrations, meteorological factors, and all-cause and cause-specific mortality. The analysis employed Generalized Additive Models (GAMs) to assess the lagged effects of O₃ exposure on mortality and to investigate the interactions between O₃ and key atmospheric determinants, including temperature, sunshine duration, and season.

**Results:**

The study revealed distinct patterns of O₃-related mortality risk modified by meteorological conditions. The 10-year average daily O₃ concentration was 92.92 μg/m^3^. O₃ exposure significantly contributed to all-cause, respiratory, and circulatory mortality with lagged effects. While atmospheric pressure, sunshine duration, temperature, and season all influenced the O₃-mortality relationship, the effect was primarily modified through significant interactions with sunshine duration, season, and temperature. These interactive health risks were more pronounced among females and the older adults.

**Conclusion:**

Our study provides strong evidence that O_3_ increases the risk of all-cause, respiratory and circulatory mortality in the population. In addition, there were interactions between meteorological factors and O_3_, primarily involving sunshine duration, season and temperature.

## 1 Introduction

Air pollution stands as the leading global environmental health risk, accounting for approximately 6.7 million deaths annually and significantly exacerbating public health crises, particularly as anthropogenic ozone (O₃) pollution continues its upward trajectory, further intensifying its role as a critical environmental determinant of the global disease burden (1, 2). In Chinese cities, O_3_ pollution has become the leading air quality concern in many urban areas (3, 4). Presently, the country faces an unparalleled crisis of O_3_ pollution, which is even more severe than in other parts of the globe (3). O_3_ concentrations in China are projected to continue rising through 2050 (5). Therefore, studying the health effects of O_3_ exposure is essential in public health research.

Ambient O_3_ pollution continues to pose a significant global environmental health hazard (6). Acute exposure drove a 94% rise in premature deaths in China during 2013–2018, and high-O₃ events boost all-cause mortality (3, 7). Nationwide cohort studies in China have shown that long-term exposure to ozone significantly increases the risk of premature death and reduces life expectancy. It harms the circulatory, cardiovascular, respiratory, and neurological systems, with delayed effects seen in higher non-accidental mortality (2, 6, 7).

Ground O_3_ levels are influenced by both anthropogenic and meteorological factors, with atmospheric parameters serving as key drivers of surface O_3_ formation in Chinese urban areas throughout the year. Multivariable regression highlighted varying impacts from hydrometeorological variables [precipitation (PE), thermal radiation (TE), relative humidity (RH), photoperiod duration (SD), barometric pressure (AP), and wind velocity (WS)], particularly thermal radiation and photoperiod duration, which are key catalysts in O_3_ photochemical processes (8). Another study found that RH, among meteorological factors, is the primary driver of changes in O_3_ concentration (9). The SD, TE difference between day and night, and extreme high and low TE are all associated with an increased risk of all-cause death among residents (10–12). Additionally, research indicates that both cold and extreme heat influence cardiovascular mortality (13); lower AP values were notably associated with the occurrence of pulmonary embolism (14), while RH and TE were associated with the mortality risk of diabetes mellitus (15). A cohort study found a notable modifying effect of TE on the relationship between mortality and O_3_ (16, 17). Extreme heat and O_3_ significantly increase daily hospitalization rates for older patients with coronary heart disease, and their synergistic interaction exhibits a dose–response relationship in exacerbating cardiovascular morbidity (18). Currently, there is no relevant research on the joint effects of other meteorological factors and O_3_ on the population’s mortality risk. As the capital of Shanxi Province, Taiyuan’s air pollution primarily stems from coal smoke, which may differ from the general patterns. Monthly urban air quality reports from China’s Ministry of Ecology and Environment show a sustained annual increase in O_3_, the primary monthly pollutant in Taiyuan, between 2015 and 2023. The association between O_3_ and all-cause mortality risk in the Taiyuan population remains unclear. Additionally, it is uncertain how O_3_ interacts with meteorological factors to influence the risk of all-cause mortality in this population. Therefore, we have conducted relevant research to provide strong evidence for controlling O_3_ levels and reducing air pollution in China and globally.

## 2 Materials and methods

### 2.1 Data collection

We collected air pollution, meteorological, and cause-of-death monitoring data from January 1, 2013, to December 31, 2022, in Taiyuan City. The air quality measurements consist of the daily average concentrations of PM_2.5_, PM_10_, SO_2_, NO_2_, and CO over 24 h, as well as the maximum 8-h average concentration of O3 (MDA8 O₃). The climatological records comprise a 24-h mean ambient temperature (°C), humidity (%), wind velocity (m/s), sunshine duration (h), rainfall accumulation (mm), and surface pressure (hPa). Comprehensive mortality figures for all causes among Taiyuan residents were sourced from the National Health Protection Information System of the Chinese Center for Disease Control and Prevention, covering January 1, 2013, to December 31, 2022. Historical meteorological data were obtained from the National Meteorological Science Data Center.^1^ Daily air pollutant data were sourced from the National Air Quality Real-time Publishing Platform.^2^ Missing values were addressed using linear interpolation. In this study, the historical observed data had a missing rate of less than 0.1%; therefore, the impact of imputation on the overall results was considered negligible.

According to the International Statistical Classification of Diseases and Related Health Problems 10th Revision (ICD-10), the daily all-cause mortality data were classified as due to tumors (C00-D48), respiratory system diseases (J00-J99), circulatory system diseases (I00-I99), and nervous system diseases (G00-G99) (19). Mortality from all causes, as well as fatalities resulting from tumors, diseases of the respiratory system, circulatory system, and nervous system, were categorized according to gender and age, defining individuals under 65 years as young and those aged 65 years and older as older adults; 24-h daily average atmospheric pressure and temperature were both dichotomized according to the median and were operationally characterized as depressed barometric pressure, elevated low barometric pressure, high barometric pressure, low temperature and high temperature in respective order. The average duration of sunshine in China is 6.39 h, based on which we divided the sunshine duration into short and long sunshine durations (20). The warm season encompasses the months of May through September, while the cold season comprises the remaining months from October to April (21). Days with temperatures at or above the 97.5 percentile are designated as “extreme heat”; all others are categorized as “non-extreme heat” (22).

### 2.2 Statistical analyses

This study used the mean, standard deviation, maximum, minimum, and quartiles to describe all-cause mortality, meteorological factors, and O_3_ in Taiyuan City from 2013 to 2022. The relationship between O_3_ and meteorological factors was analyzed using Pearson’s correlation and corresponding heat maps. We conducted quantitative modelling to establish the concentration-mortality associations between ambient O_3_ exposure and population-wide mortality outcomes, deaths due to respiratory diseases, circulatory diseases, neurological diseases, and tumors, respectively, using generalized additive models (GAMs), which controlled for long-term trends, “the day of week (DOW)” effect, and environmental determinants. Dose–response associations were subjected to stratified analytical evaluation incorporating gender-specific and age-cohort variables. We conducted lag (lag0-lag3) and cumulative lag (lag01-lag03) analyses to adjust for possible lagged impacts. Based on the results of the correlation analysis, the effects of meteorological factors on the relationships between O_3_ and all-cause deaths, deaths due to respiratory diseases, circulatory diseases, neurological diseases, and tumors were analyzed using GAMs with hierarchical parameters. The lagged and cumulative lagged effects of O_3_ were also investigated. An overdispersed Poisson regression model was employed. The models are as follow Equation 1:


$$\begin{array}{l} \log[E(Y_{t})]=\alpha +\beta_{1}(X)+\beta_{2}(M_{k})+\beta_{3}(X:M_{k})\\ +{\sum^{p}}_{j=1}\mathrm{fj}(\mathrm{Zj},\mathrm{df})+W_{t}(\mathrm{DOW}) \end{array}$$ (1)

*X* represents the concentration of O_3_; *M_k_* represents dichotomized meteorological factors; *β*_1_*(X)* represents the effect of O_3_ when the meteorological factor is the reference category; *β*_2_*(M_k_)* represents the effect when the meteorological factor is in another category; *β*_3_(*X*: *M_k_*) represents the interaction effect of O_3_ and meteorological factors; $\sum_{j=1}^{p}\mathrm{fj}(\mathrm{Zj},\mathrm{df})$ is the non-parametric spline function of other variables including death date, meteorological factors, and other air pollutants. Natural cubic splines with 3 degrees of freedom were used to smooth meteorological factors and air pollutants, while natural cubic splines with 7 degrees of freedom were employed to control for long-term temporal trends. *Wt*(*DOW*) is a dummy variable for the day of the week. The effect of O_3_ when the meteorological factor is another category is *β*_1+_*β_3_*, and the odds ratio (*OR*) and corresponding 95% confidence interval are calculated. We used the “mgcv” package from R 4.0.2 software. A *p* < 0.05 was considered statistically significant. Model specification was selected based on the Akaike Information Criterion for the quasi-Poisson model (Q-AIC), while variable selection was consistent with previous studies (23, 24).

## 3 Results

### 3.1 Description of mortality, O_3d_ and meteorological factors in Taiyuan

**Figure 1:**
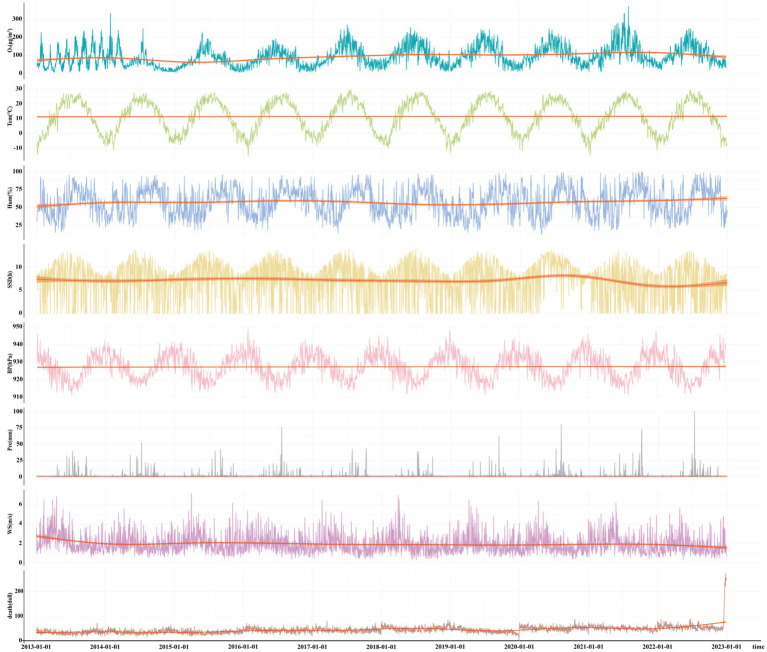
Temporal changes of O_3_, meteorological factors and all-cause deaths in Taiyuan from 2013 to 2019.

Table 1 shows the mortality rate in Taiyuan from 2013 to 2022. The results show that the all-cause mortality rate (7.72–13.79%), respiratory disease mortality rate (7.71–12.99%), circulatory disease mortality rate (7.65–14.50%), nervous system disease mortality rate (5.99–18.13%), and tumor mortality rate (8.22–12.33%) in Taiyuan from 2013 to 2022 exhibit an increasing trend. Table 2 included the number of daily deaths, O_3_ and meteorological factors, with 20.84, 12.15 and 3.82 deaths per day from circulatory diseases, tumors and respiratory diseases, respectively, being the top three. MDA8 O₃, temperature, relative humidity, precipitation, barometric pressure, wind speed and sunshine duration are 92.92 μg/m^3^, 11.37 °C, 57.13%, 2.59 mm, 927.24 hPa, 1.95 m/s and 7.11 h during the period from 2013 to 2022, respectively. Figure 1 illustrates the temporal changes in O_3_, meteorological factors, and all-cause deaths over time in Taiyuan from 2013 to 2022.

**Table 1:** Annual deaths in Taiyuan from 2013 to 2022 [*n* (%)].

Years	Overall deaths	Respiratory system disease	Circulation system disease	Nervous system disease	Tumor
2013	12,504 (7.72)	1,075 (7.71)	5,820 (7.65)	107 (5.99)	3,647 (8.22)
2014	13,179 (8.14)	1,383 (9.92)	5,986 (7.87)	115 (6.44)	3,705 (8.35)
2015	12,445 (7.69)	1,377 (9.87)	5,521 (7.26)	94 (5.26)	3,624 (8.17)
2016	15,673 (9.68)	1,455 (10.43)	7,261 (9.54)	151 (8.45)	4,406 (9.93)
2017	15,725 (9.71)	1,539 (11.04)	7,473 (9.82)	154 (8.62)	4,393 (9.90)
2018	18,256 (11.27)	1,688 (12.10)	8,477 (11.14)	187 (10.46)	4,948 (11.15)
2019	14,654 (9.05)	1,256 (9.01)	6,422 (8.44)	169 (9.46)	4,270 (9.62)
2020	18,628 (11.50)	1,144 (8.20)	9,112 (11.97)	229 (12.81)	5,033 (11.34)
2021	18,550 (11.45)	1,217 (8.73)	8,988 (11.81)	257 (14.38)	4,877 (10.99)
2022	22,325 (13.79)	1811 (12.99)	11,031 (14.50)	324 (18.13)	5,472 (12.33)

**Table 2:** MDA8 O₃, meteorological factors and death toll in Taiyuan from 2013 to 2022.

Variables	Mean	SD	Min	*P_25_*	*P_50_*	*P_75_*	Max
MDA8 O₃ (μg/m^3^)	92.92	55.15	3.69	51.29	82.86	125.71	371.71
Tem (°C)	11.37	10.58	−14.90	1.80	12.30	21.00	29.60
Hum (%)	57.13	18.93	12.00	42.00	57.00	72.00	100.00
Pre (mm)	2.59	2.61	0.00	0.00	2.44	4.44	9.95
BP (hPa)	927.24	7.00	911.40	921.20	927.60	932.60	948.90
WS (m/s)	1.95	0.99	0.30	1.20	1.70	2.40	7.20
SSD (h)	7.11	3.87	0.00	4.70	8.10	10.00	13.80
Overall deaths	44.34	15.57	3	35	43	52	271
Respiratory system disease	3.82	2.97	0	2	3	5	59
Circulation system disease	20.84	8.79	0	15	20	25	141
Nervous system disease	0.49	0.74	0	0	0	1	6
Tumor	12.15	4.15	1	9	12	15	40

### 3.2 Analysis of the correlation between O_3_ and meteorological factors in Taiyuan

**Figure 2:**
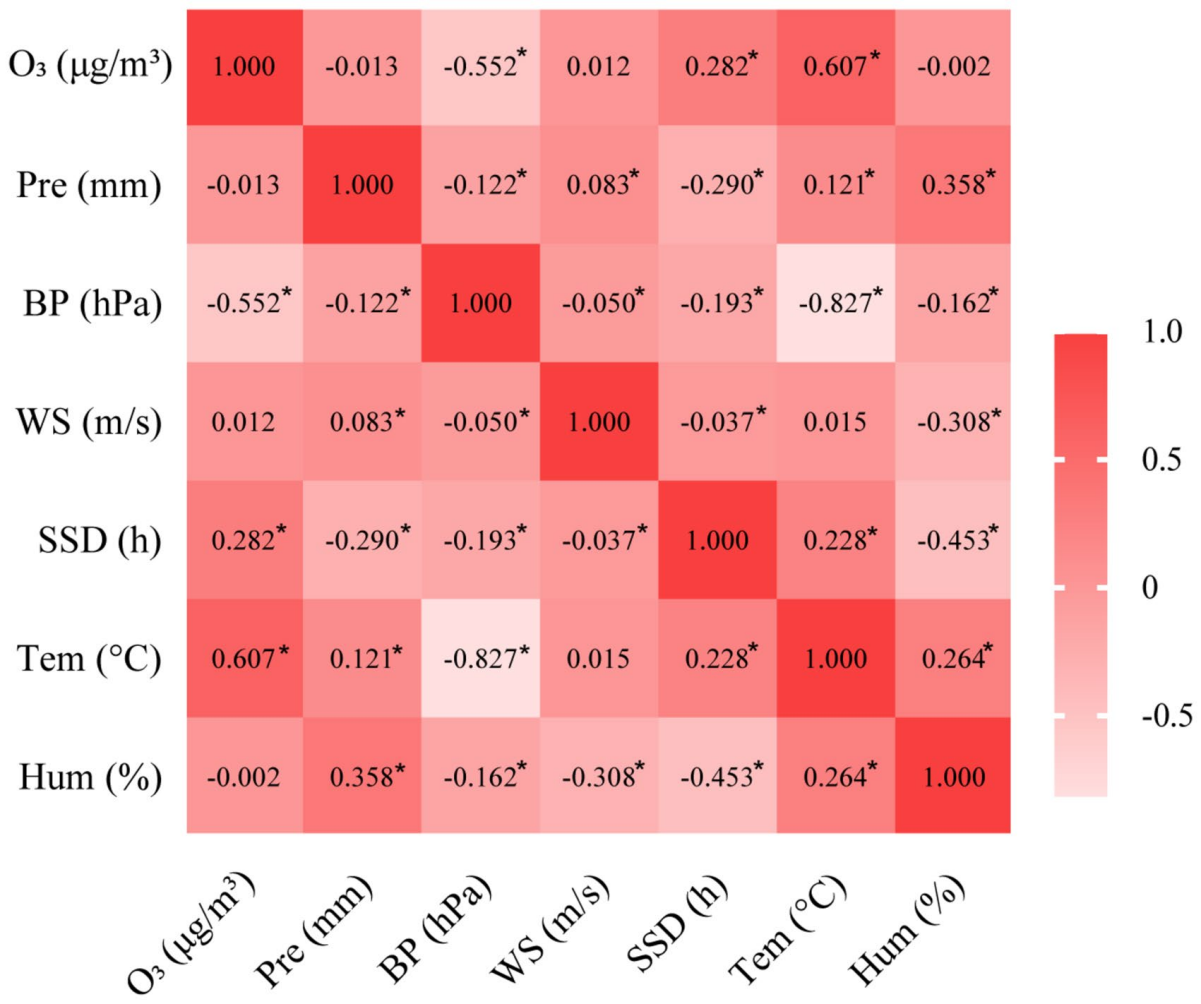
Correlation heat map between O_3_ and meteorological factors.

Our study used Pearson’s correlation to analyze the correlation between O_3_ and meteorological factors. Statistical analyses revealed ambient O_3_ concentrations demonstrated statistically significant positive associations with ambient thermal metrics and sunshine duration (Pearson’s r coefficients = 0.607, 0.282) while exhibiting inverse correlations with atmospheric pressure measurements (r = −0.552), but not strongly correlated with relative humidity, wind speed, and precipitation, as detailed in Figure 2.

### 3.3 All-cause mortality risk and stratified analysis in Taiyuan

**Figure 3:**
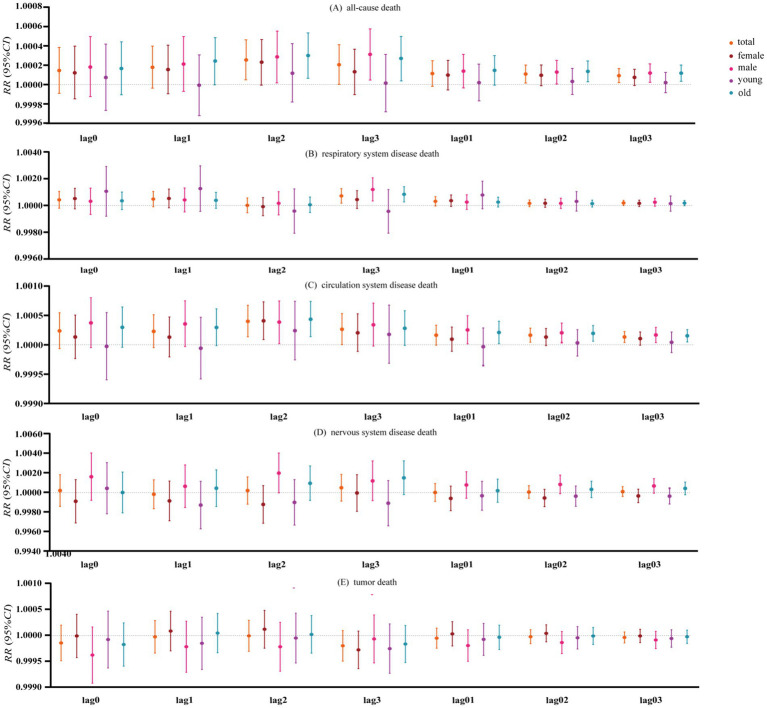
Risk of all-cause death and stratified risk map of causes.

Figure 3 shows the death risk of O_3_ from lag0 to lag03 and the death risk after stratification by gender and age, respectively. The analytical findings demonstrate that ambient O_3_ exposure constitutes a non-negligible risk factor for elevated mortality hazards across all causes, mortality from respiratory diseases and mortality from circulatory diseases. In the all-cause mortality risk for the whole population, men and older population, the risk of death for lag2_O_3_ was 1.000254 (95% *CI*: 1.000048–1.000460), 1.000286 (95% *CI*: 1.000016–1.000556) and 1.000300 (95% *CI*: 1.000064–1.000537); the risk of death for lag3_O_3_ was 1.000205 (95% *CI*: 1.000002–1.000408), 1.000312 (95% *CI*: 1.000046–1.000578) and 1.000270 (95% *CI:* 1.0000374–1.000504); the risks of death for lag02_O_3_ were 1.000109 (95% *CI*: 1.000015–1.000202), 1.000128 (95% *CI*: 1.000006–1.000251) and 1.000136 (95% *CI*: 1.0000282–1.000243); the risks of death for lag03_O_3_ were 1.0000918 (95% *CI*: 1.000019–1.000164), 1.000118 (95% *CI*: 1.0000228–1.000213) and 1.000117 (95% *CI*: 1.0000337–1.000201), respectively (Figure 3A). The risk of death from respiratory diseases in the whole population, men and the older population, was 1.000711 (95% *CI*: 1.000167–1.001254), 1.001189 (95% *CI*: 1.000332–1.002046) and 1.000831 (95% *CI*: 1.000263–1.001399) for lag3_O_3_, respectively (Figure 3B). In the risk of death due to circulatory disease for the whole population, women, men and the older population, the risk of death for lag2_O_3_ was 1.000399 (95% *CI*: 1.000136–1.000663), 1.000408 (95% *CI*: 1.000088–1.000728), 1.000386 (95% *CI*: 1.000019–1.000752), and 1.000434 (95% *CI*: 1.000139–1.000729), respectively. The overall mortality risk due to circulatory disease across the population indicated that the risk associated with lag3_O_3_ was 1.000264 (95% *CI*: 1.000003–1.000524). For lag01_O_3_, the mortality risk from circulatory disease was found to be 1.000253 (95% *CI*: 1.000016–1.000489) for the general population, with values of 1.000209 (95% *CI*: 1.000018–1.000400) explicitly noted for males and older adults. In the risk of death due to circulatory diseases in the whole population, male and older population, the risk of death for lag02_O_3_ was 1.000163 (95% *CI*: 1.000044–1.000283), 1.000204 (95% *CI*: 1.000038–1.000370), and 1.000193 (95% *CI*: 1.000059–1.000327), and for lag03_O_3_ was 1.000132 (95% *CI*: 1.000039–1.000225), 1.000167 (95% *CI*: 1.0000374–1.000296) and 1.000153 (95% *CI*: 1.000048–1.000257) (Figure 3C). O_3_ had no significant effect on the population’s risk of death from neurological diseases and death from tumors (Figures 3D,E).

### 3.4 Analysis of the interaction between O_3_ and meteorological factors in Taiyuan

**Figure 4:**
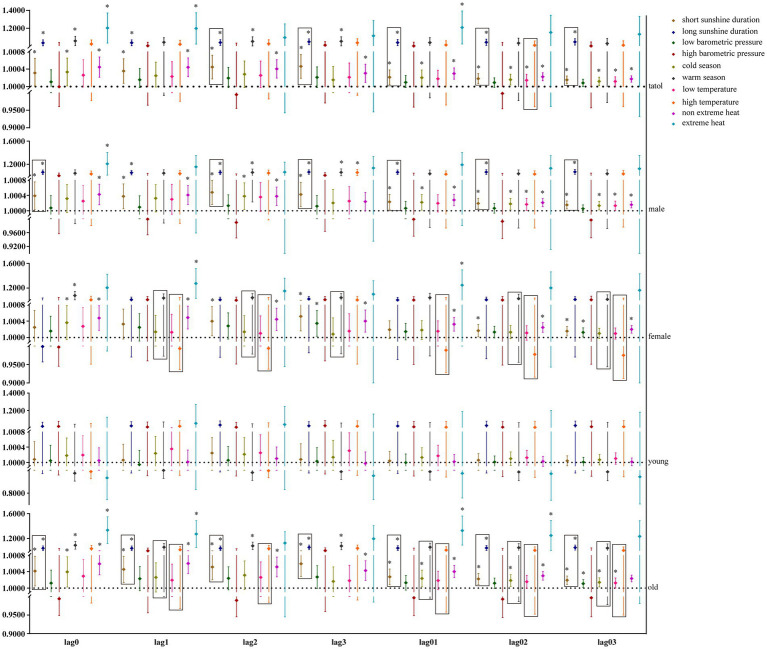
Interaction effect of all-cause death risk. Rectangles represent variables with interaction effects.

**Figure 5:**
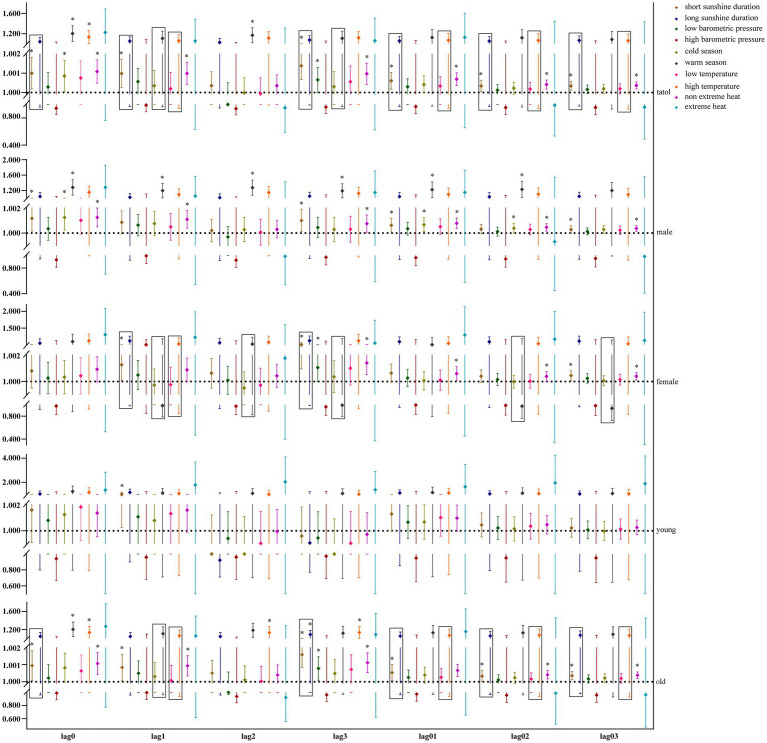
Interaction effect of respiratory system disease death risk. Rectangles represent variables with interaction effects.

**Figure 6:**
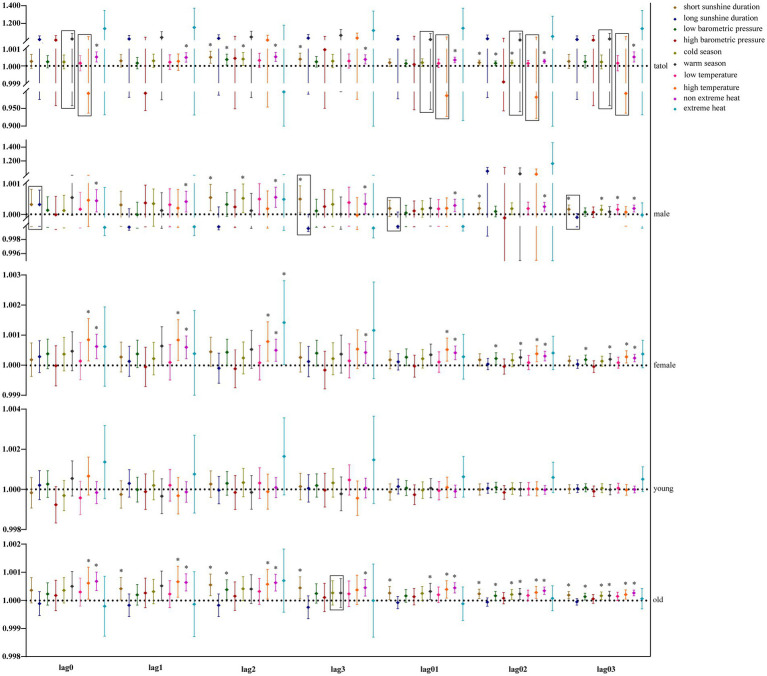
Interaction effect of nervous system disease death risk. Rectangles represent variables with interaction effects.

**Figure 7:**
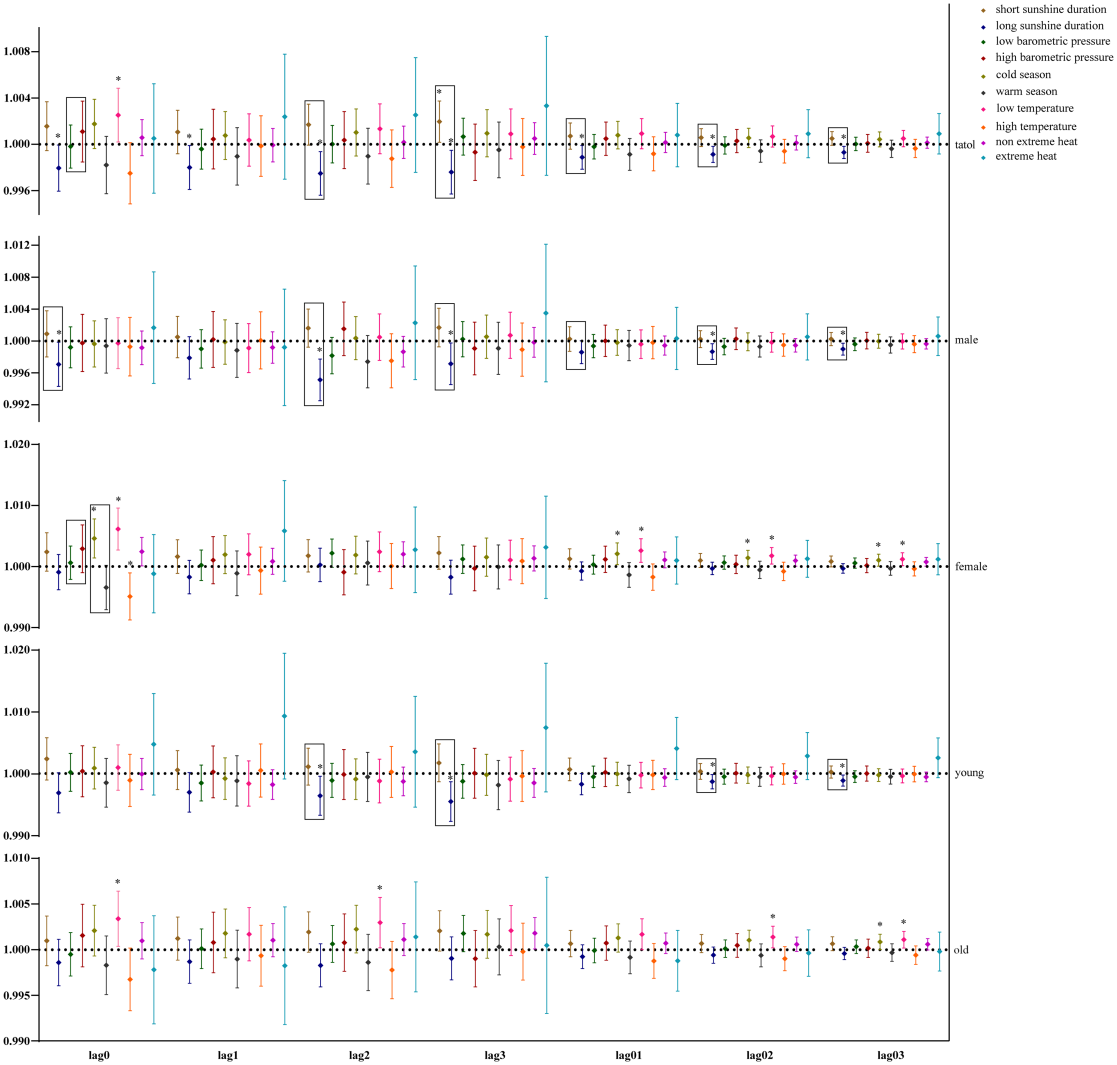
Interaction effect of nervous system disease death risk. Rectangles represent variables with interaction effects.

**Figure 8:**
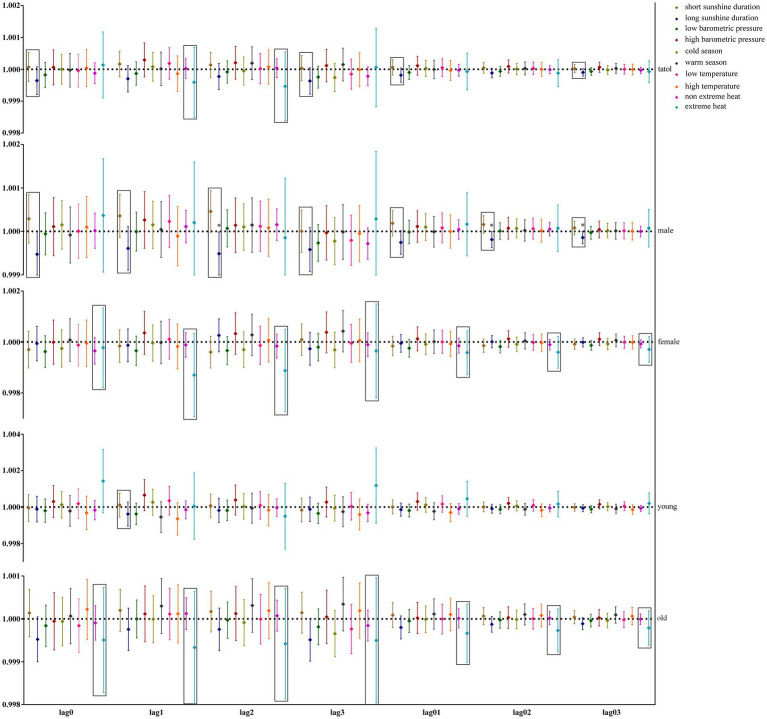
Interactive effect of tumor death risk. Rectangles represent variables with interaction effects.

Our study further utilized GAM with stratification parameters to analyze the mortality risk associated with O_3_ and the interaction between O_3_ and meteorological factors. Figure 4 illustrates the impact of O_3_ on the risk of all-cause mortality across the entire population, including men, women, and the older adults, in relation to various meteorological factors, with O_3_ also interacting with hours of sunlight. O_3_ is more likely to interact with seasons and temperature in women and the older adults. Figure 5 illustrates the impact of O_3_ on the risk of death from respiratory diseases in the entire population, as well as in men, women, youth, and the older adults, considering various meteorological factors. O_3_ interacts with the duration, season, and intensity of sunlight across the whole population, including women and the older adults. Figure 6 shows the impact of O_3_ on the risk of death from circulatory diseases in the entire population, men, women and the older adults, with different meteorological factors, with O_3_ interacting with sunshine duration in men, while O_3_ interacts with both season and temperature in the population as a whole, and with season in the older adults. Figure 7 shows the impact of O_3_ on the risk of death from neurological diseases in the whole population, males, females, youth and the older adults, with different meteorological factors, with O_3_ interacting with sunshine duration in the whole population, males and youth; and O_3_ interacting with atmospheric pressure in the whole population and females, and also with season in females. Figure 8 illustrates the effect of O_3_ on the risk of death from tumor in males, considering various meteorological factors. O_3_ interacts with sunshine duration for the entire population, including men and young people, as well as with the presence or absence of weather extremes for the entire population, women, and the older adults.

## 4 Discussion

The results of our study show that the number of deaths in Taiyuan City from 2013 to 2022 exhibits a trend of slow increase year by year, which is consistent with the trend of steady increase in O_3_ in China as one of the air pollutants (1). Therefore, exploring the relationship between O_3_ and mortality risk in Taiyuan City is essential. Although the average daily O_3_ concentration in Taiyuan from 2013 to 2022 is 92.92 μg/m3, which is lower than China’s primary limit (100 μg/m3) and secondary limit (160 μg/m3), the time series shows periodic fluctuations. There are more periods when the O_3_ level is higher than China’s primary and secondary limits. We suggest that paying attention to O_3_ pollution levels in Taiyuan is essential to establish an early warning system and strengthen O_3_ prevention and control. Our study reveals significant correlations among O₃ levels, temperature, sunshine duration, and barometric pressure, likely driven by year-round influences of these meteorological factors as primary contributors to ground-level O₃ formation. Our findings align with prior studies, indicating that climatic parameters such as precipitation, humidity, and wind speed variably affect O₃ levels, with temperature/solar radiation, and relative humidity identified as key determinants in separate analyses (8, 9). Therefore, it is crucial to mitigate O_3_-related health risks by investigating the influence of meteorological factors on O_3_ levels and population mortality.

It is generally accepted that O_3_ has a lagged effect on population mortality. Our results show that O_3_ has a lag in the risk of all-cause mortality and in the risk of death due to respiratory disease, with gender- and age-stratified results showing that the lag is still present in males and older age groups. An epidemiological study found that a 10 μg/m^3^ increase in 24-h lagged O_3_ exposure correlates with a statistically significant 1.38% increase in population mortality risk (25). A longitudinal study linked a 10 μg/m^3^ rise in 3-day cumulative O_3_ exposure to a 0.24% increase in population mortality (26). The results show that for every 10 μg/m^3^ increase in 2-day lagged ambient O₃ exposure, the risk of all-cause mortality increases by 0.0254% (*p* < 0.05), with comparable risk magnitudes showing non-significant variation. Furthermore, the analytical framework quantified a statistically significant 0.0711% increase in respiratory disease-specific mortality per 10 μg/m^3^ rise in 3-day cumulative O₃ exposure (*p* < 0.01), consistent with prior correlational studies identifying lag3 O₃ as the exposure window with the highest respiratory mortality risk (27). A peer-reviewed study reported a statistically significant 0.09% elevation in respiratory disease mortality per 10 μg/m^3^ 24-h lagged O_3_ exposure (*p* < 0.05). In comparison, a comparable epidemiological investigation demonstrated a 0.78% increase in respiratory mortality burden with 3-day cumulative O_3_ exposure (*p* < 0.01) (2, 27), which was similar to our study. This may be because O_3_ entering the respiratory system can exacerbate a series of responses, such as oxidative stress, inflammation, and lung injury, ultimately leading to worsening respiratory disease and death (28–30). Our analysis revealed a lagged association between ambient O_3_ exposure and circulatory disease mortality, persisting across gender and age subgroups. The highest risk occurred at lag 2 O_3_ exposure, with a 0.0399% increase in circulatory mortality per 1 μg/m^3^ rise (*p* < 0.05). Stratified analyses showed stronger female susceptibility (0.0408% vs. 0.0386% in males) and greater older adults vulnerability (0.0434% increase). A prior study reporting 0.11% mortality elevation per 10 μg/m^3^ lag01 O_3_ exposure yielded lower risk estimates than our findings (2). Still, our study spanned a considerably more extended period; the results are likely to be more realistic. It also noted that females and the older adults were more susceptible to O_3_ exposure. This is mainly because O_3_ enters the circulatory system through the blood-oxygen barrier after entering the lungs via the respiratory tract, causing vascular inflammation, oxidative damage, and vascular endothelial dysfunction (31, 32).

Meteorological factors play a crucial role in the risk of death among O_3_-affected populations. Our study reveals that meteorological factors contribute to the risk of O_3_-induced mortality from all-cause, respiratory, circulatory, and neurological diseases, including sunshine duration, season, temperature, barometric pressure, and extreme heat, with sunshine duration, season, and temperature being the primary factors. Nonetheless, the impact of O_3_ on tumor mortality risk was unaffected by meteorological factors. Currently, there are inconsistent findings on the effect of O_3_ on tumor mortality, with one study showing no significant correlation between O_3_ and the risk of death due to malignant tumors (33). In contrast, another study showed that O_3_ increases lung cancer mortality and that warm and cold seasons play an important role in this effect (34). Research on the interplay between O_3_ and meteorological factors in relation to tumor mortality risk remains sparse, indicating a need for further investigation. Our study, after categorizing participants by gender and age, revealed that the duration of sunshine influences the risk of mortality from tumors associated with O_3_ in males.

In summary, sunshine duration emerged as the primary modifier of O_3_-related mortality risk, with significant interactions observed between O_3_ exposure and meteorological factors, including barometric pressure, season, temperature, and extreme heat. Our findings align with studies demonstrating temperature-O_3_ synergies in ischemic heart disease pathogenesis (35) and elevated warm-season O_3_-associated mortality (17, 36). In general, long sunshine duration, high temperature and warm season contribute to O_3_’s increased mortality risk.

Our study provides scientific evidence for mitigating O_3_-related mortality risks by integrating meteorological factors into O_3_ mortality risk assessments, with a particular emphasis on sunshine duration as a key modifier. While findings support integrating meteorological data into O_3_ mitigation strategies, limitations persist—notably, the existing literature underscores that extreme heat amplifies O_3_-associated cardiovascular mortality in populations under 65, a gap warranting further investigation (28, 37). The frequency of extreme heat events in the dataset of this study was limited; thus, further expansion of the dataset is required to explore the role of extreme heat events in ozone-related mortality risk. Future work will expand the dataset to further investigate these interactions. This study provides a robust scientific foundation for informing O_3_ control strategies and mitigating population-level mortality risks associated with O_3_ exposure. Nevertheless, it is worth noting that this study has other limitations. Firstly, the collection of pollutant data relies on fixed monitoring stations, which may introduce exposure measurement bias; this is an inherent limitation in most studies (23, 38).

## 5 Conclusion

Our study provides strong evidence that O_3_ increases the risk of all-cause, respiratory and circulatory mortality in the population. In addition, there were interactions between meteorological factors and O_3_, primarily involving sunshine duration, season, and temperature.

## Data Availability

The data analyzed in this study is subject to the following licenses/restrictions: the datasets generated and/or analyzed during the current study are not publicly available due to data ownership by a third-party institution, but are available from the corresponding author upon reasonable request. Requests to access these datasets should be directed to ZZ, zzh1973@sxmu.edu.cn.

## Footnotes

1
https://data.cma.cn/

2
https://air.cnemc.cn:18007/
